# Clinical and epidemiological characteristics of 96 pediatric human metapneumovirus infections in Henan, China after COVID-19 pandemic: a retrospective analysis

**DOI:** 10.1186/s12985-024-02376-0

**Published:** 2024-04-30

**Authors:** Wangquan Ji, Yu Chen, Shujie Han, Bowen Dai, Kang Li, Shuang Li, Zijie Li, Shouhang Chen, Yaodong Zhang, Xiaolong Zhang, Xiaolong Li, Qingmei Wang, Jiaying Zheng, Chenyu Wang, Qiujing Liang, Shujuan Han, Ruyu Zhang, Fang Wang, Yuefei Jin

**Affiliations:** 1 Department of Infectious Diseases, Children’s Hospital Affiliated to Zhengzhou University, Henan Children’s Hospital, Zhengzhou Children’s Hospital, Zhengzhou, 450018 Henan China; 2 Department of Epidemiology, College of Public Health, Zhengzhou University, Zhengzhou, 450001 Henan China; 3 Henan International Joint Laboratory of Children’s Infectious Diseases, Children’s Hospital Affiliated to Zhengzhou University, Henan Children’s Hospital, Zhengzhou Children’s Hospital, Zhengzhou, 450018 Henan China; 4 NHC Key Laboratory of Birth Defects Prevention; Henan Key Laboratory of Population Defects Prevention, Zhengzhou, 450002 Henan China

**Keywords:** Human metapneumovirus, Pneumonia, Epidemiological characteristics, Clinical characteristics, Coinfection

## Abstract

**Background:**

In the aftermath of the COVID-19 pandemic, there has been a surge in human metapneumovirus (HMPV) transmission, surpassing pre-epidemic levels. We aim to elucidate the clinical and epidemiological characteristics of HMPV infections in the post-COVID-19 pandemic era.

**Methods:**

In this retrospective single-center study, participants diagnosed with laboratory confirmed HMPV infection through Targeted Next Generation Sequencing were included. The study encompassed individuals admitted to Henan Children's Hospital between April 29 and June 5, 2023. Demographic information, clinical records, and laboratory indicators were analyzed.

**Results:**

Between April 29 and June 5, 2023, 96 pediatric patients were identified as infected with HMPV with a median age of 33.5 months (interquartile range, 12 ~ 48 months). The majority (87.5%) of infected children were under 5 years old. Notably, severe cases were statistically younger. Predominant symptoms included fever (81.3%) and cough (92.7%), with wheezing more prevalent in the severe group (56% *vs* 21.1%). Coinfection with other viruses was observed in 43 patients, with Epstein–Barr virus (EBV) (15.6%) or human rhinovirus A (HRV type A) (12.5%) being the most common. Human respiratory syncytial virus (HRSV) coinfection rate was significantly higher in the severe group (20% *vs* 1.4%). Bacterial coinfection occurred in 74 patients, with Haemophilus influenzae (Hin) and Streptococcus pneumoniae (SNP) being the most prevalent (52.1% and 41.7%, respectively). Severe patients demonstrated evidence of multi-organ damage. Noteworthy alterations included lower concentration of IL-12p70, decreased lymphocytes percentages, and elevated B lymphocyte percentages in severe cases, with statistical significance. Moreover, most laboratory indicators exhibited significant changes approximately 4 to 5 days after onset.

**Conclusions:**

Our data systemically elucidated the clinical and epidemiological characteristics of pediatric patients with HMPV infection, which might be instructive to policy development for the prevention and control of HMPV infection and might provide important clues for future HMPV research endeavors.

**Supplementary Information:**

The online version contains supplementary material available at 10.1186/s12985-024-02376-0.

## Introduction

Human metapneumovirus (HMPV), a member of paramyxovirus family, was first identified in 2001 [1]. It has since been commonly implicated in acute respiratory tract infections (ARTI) affecting both pediatric and adult populations worldwide. Despite efforts, a live-attenuated recombinant HMPV vaccine has demonstrated inadequate immunogenicity in children aged 6–59 months [2], and to date, no licensed vaccines or specific drugs are available for HMPV infections. Primary infections typically occur before the age of 5 years, with HMPV prevalence among this age group ranging from 1.1% to 86% globally [3]. In 2018, HMPV-associated hospital admissions among children under 5 years old globally amounted to 643,000, with 16,100 (hospital and community) HMPV-associated ARTI deaths [4]. These figures underscore the substantial socio-economic impact and disease burden associated with HMPV infection.

Over the past 3 years, the unprecedented implementation of non-pharmaceutical interventions during the COVID-19 pandemic has significantly impacted the epidemiology of various pediatric infectious diseases [5–7]. The return of respiratory virus circulation to pre-pandemic levels is anticipated as COVID-19 mitigation measures gradually ease [8]. The concept of “immunity debt” has been proposed to characterize the paucity of protective immunity resulting from prolonged decreased exposure to various pathogens [9]. In Western Australia, the incidence of HMPV infection surged threefold in 2021 compared to the period of 2017 ~ 2019. Moreover, the proportion of respiratory-coded admissions undergoing HMPV testing doubled in 2021 [10, 11]. Similarly, in the United States, the number of HMPV infections suddenly spiked to record levels in the spring of 2023 (https://www.cdc.gov/surveillance/nrevss/hmpv/region.html). In China, several studies conducted before the COVID-19 pandemic have examined the prevalence and genotypic diversity of HPMV, as well as the epidemiological and clinical characteristics of hospitalized patients with HPMV infection [12–16]. A study conducted in the Netherlands demonstrated that the clinical impact of HMPV infection remained consistent between the non-COVID-19 period and the examined COVID-19 period, with no significant changes observed in terms of incidence and/or disease severity [17]. Nevertheless, the epidemiological and clinical characteristics of HMPV infections have shown disparities following the COVID-19 pandemic. Consequently, there is an urgent need for clinical investigations, particularly focusing on children hospitalized with HMPV infection, to provide a reference for clinical diagnosis and management. In this study, we present a detailed analysis of the clinical and epidemiological features of 96 pediatric patients in central China from April 29 to June 5, 2023. Our findings contribute to a comprehensive understanding of the characteristics of HMPV infections in the post-COVID-19 pandemic era.

## Materials and methods

### Study design and participants

**Fig. 1:**
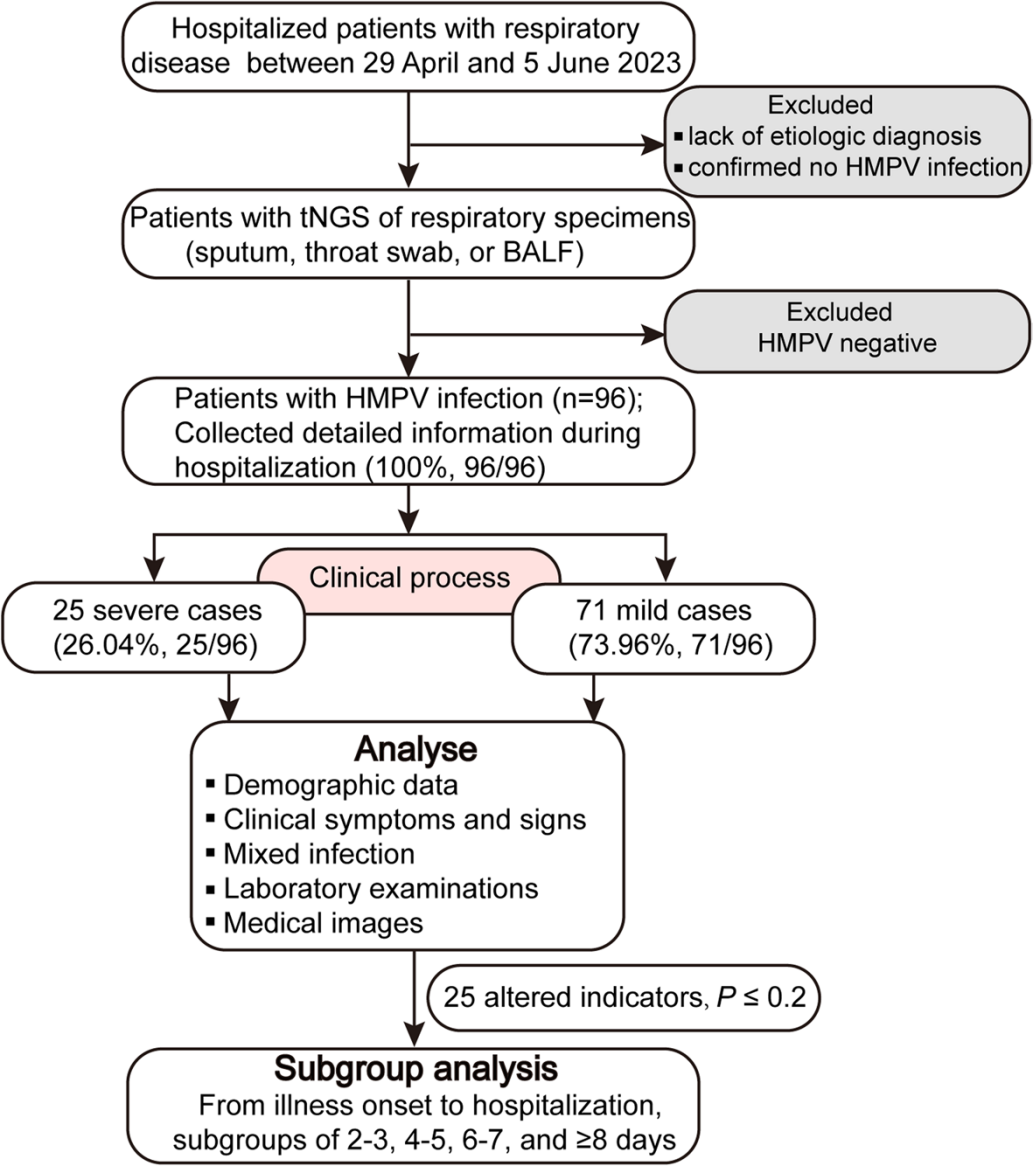
Flow chart of participants enrolment and data analysis procedure in the study. BALF, bronchoalveolar lavage fluid

For this retrospective study, we included a total of 96 hospitalized pediatric patients diagnosed with HMPV infection who were admitted to Children’s Hospital Affiliated to Zhengzhou University between 29 April and 5 June 2023 (Fig. 1). Children’s Hospital Affiliated to Zhengzhou University (Henan Children’s Hospital) is the largest tertiary pediatric referral hospital in central China with 2,200 beds, which is located in Zhengzhou of Henan province. Respiratory specimens (including sputum, throat swab, or bronchoalveolar lavage fluid (BALF)) from the majority of hospitalized patients with respiratory illness underwent testing for respiratory pathogens using Targeted Next Generation Sequencing (tNGS) conducted by Guangzhou Kingmed Ctr for Clin Lab Co ltd. (https://www.kingmed.com.cn/) [18]. All enrolled patients were categorized as either mild or severe based on the guidelines for the management of community-acquired pneumonia in children of the People’s Republic of China (2013 Edition) [19, 20]. This observational study was approved by the Committee for Ethical Review of Zhengzhou University (ethical approval No: ZZUIRB2023-180), and written informed consent was obtained from the parents.

### Data collection

The electronic medical records data of the included patients underwent independently review and retrospective collection by trained researchers. To ensure quality control, two additional researchers cross-checked the data collection forms. Detailed information was extracted, encompassing sequencing data, demographic data, clinical symptoms and signs, laboratory examinations, medical images, and outcomes of treatment. Laboratory examinations comprised routine testing, coagulation function tests, lymphocyte subsets, inflammatory or infection-related biomarkers, analysis of immunological responses, and measurement of biomarkers for monitoring liver, myocardial, and renal functions.

### Statistical analysis

The extracted data were initially entered into Microsoft Excel software (2016), and subsequently imported into SPSS 25.0 or GraphPad Prism 8.3 software for statistical analysis. Binomial or categorical variables were expressed as percentages, while clinical characteristics and laboratory findings (continuous variables) were presented as median with interquartile range (IQR). To compare variables across groups, the Mann–Whitney test was used for continuous variables, and Chi-squared test or Fisher exact test was employed for categorical variables. All statistical tests were two-sided, and a *P*-value < 0.05 was considered statistically significant.

## Results

### Epidemiology and demographic characteristics of HPMV infections

**Fig. 2:**
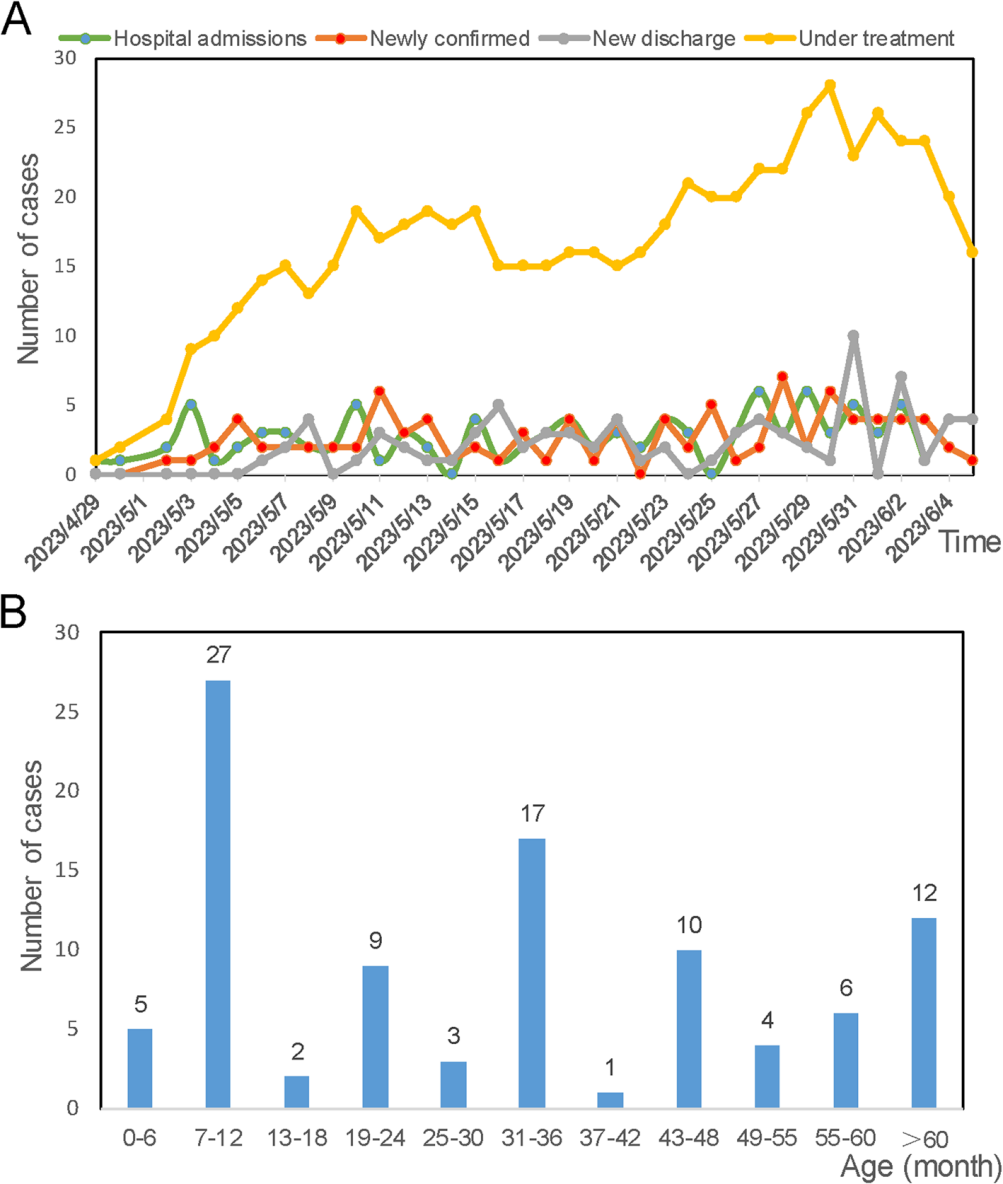
Time of onset and age distribution of laboratory-confirmed HMPV infections Clinical signs of HMPV infections

HMPV infections were almost detected and hospitalized every day between April 29 and June 5, 2023. The number of infected patients under treatment is highest at May 30 (28 patients), and 16 infected patients remained hospitalized at June 5 (Fig. 2A). In total, 96 pediatric patients (25 severe cases and 71 mild cases), were included in this study, with a median age of 33.5 months (IQR: 12–48 months). Sequencing data (partial M and N gene) from 9 patients were randomly selected and subjected to analysis. Detailed sequencing data are provided in Supplementary Fig. S1. Through multiple alignments with the nearest homologies from the NCBI databases, it was determined that all the selected viruses belong to subtype A2b. The majority (87.5%, 84/96) of infected children were under 5 years old, and more than half (65.63%, 63/96) were under 3 years old (Fig. 2B). As shown in Table 1, children in the severe group tended to be younger, with a median age of 1 year, compared to a median age of 3 years in the mild group. The age distribution significantly differed between the severe and mild groups (*P* = 0.005). The male to female ratio was 1.53 (58/38). Although the difference was not significant (*P* = 0.06, 77.5% *vs* 48%), a higher proportion of patients in the mild group resided in urban areas.

**Table 1:** Demographic characteristics and Clinical symptoms, signs of HMPV infections

	**All patients** **(No. = 96)**	**Mild pneumonia** **(No. = 71)**	**Severe pneumonia** **(No. = 25)**	***P* value**
**Demographics**	--	--	--	--
Age, median (IQR), months	33.5 (12.0, 48.0)	36 (12.0, 53.0)	12 (7.5, 36.0)	0.005
Male sex, No. (%)	58 (60.4)	42 (59.2)	16 (64.0)	0.670
Urban, No. (%)	67 (69.8)	55 (77.5)	12 (48.0)	0.06
**Symptoms on presentation**	--	--	--	--
Fever, No. (%)	78 (81.3)	60 (84.5)	18 (72.0)	0.168
Cough, No. (%)	89 (92.7)	66 (93.0)	23 (92.0)	0.874
Wheezing, No. (%)	29 (30.2)	15 (21.1)	14 (56.0)	0.001
Fever, cough, wheezing, No. (%)	17 (17.7)	9 (12.7)	8 (32.0)	0.063
Fever, cough, No. (%)	55 (57.3)	47 (66.2)	8 (32.0)	0.003
Fever, wheezing, No. (%)	2 (2.1)	2 (2.8)	0 (0.0)	1.000
Cough, wheezing No. (%)	10 (10.4)	4 (5.6)	6 (24.0)	0.018
Fever alone, No. (%)	4 (4.2)	2 (2.8)	2 (8.0)	0.277
Cough alone, No. (%)	7 (7.3)	6 (8.5)	1 (4.0)	0.672
**Vital signs at presentation**	--	--	--	--
Temperature, median (IQR), No., °C	36.8 (36.5, 37.2), 96	36.8 (36.5, 37.1), 71	37.0 (36.7, 37.8), 25	0.133
Temperature max, median (IQR), No., °C	39.0 (38.5, 39.5), 86	39.0 (38.5, 39.6), 65	38.8 (38.4, 39.5), 21	0.636
Respiratory rate, median (IQR), No., breaths/min	28.0 (25.0, 32.0), 96	26.0 (25.0, 30.0), 71	35.0 (28.0, 44.5), 25	0.000003
Heart rate, median (IQR), No., /min	117.0 (106.5, 130.0), 96	112.0 (105.0, 124.0), 71	130.0 (114.0, 163.5), 25	0.000191
Weight, median (IQR), No., Kg	13.1 (10.4, 18.0), 94	14.0 (11.7, 18.0), 69	11.0 (8.8, 14.6), 25	0.002
Birth weight, median (IQR), No., Kg	3.3 (3.0, 3.5) 93	3.3 (3.1, 3.4), 69	3.3 (3.0, 3.5), 24	0.975
Duration from illness onset to hospitalization, median (IQR), days	5.0 (4.0, 9.0)	5.0 (4.0, 11.0)	4.0 (3.0, 7.5)	0.112
Duration from hospitalization to diagnosis, median (IQR), days	2 (1.0, 2.0)	2 (1.0, 2.0)	2 (1.0, 2.0)	0.346
Duration from diagnosis to hospital discharge, median (IQR), days	4 (2.0, 9.0)	4 (3.0, 5.0)	6 (4.5, 9.5)	0.000027
Duration of hospitalization, median (IQR), days	6.0 (5.0, 8.0)	6.0 (5.0, 7.0)	8.0 (6.0, 13.5)	0.000321

Categorical variables are expressed as numbers (%), and continuous variables as median (IQR). 4 special cases: 2 patients were hospitalized without respiratory symptoms as the main complaint, including a patient with burns and a patient with gastrointestinal bleeding. After admission, diagnosis of hemophagocytic syndrome was highly suspected in a patient with deteriorated condition. Additionally, a patient was diagnosed with ganglioneuroblastoma

In Table 1, we present a summary of the characteristics observed in HMPV infections. Upon admission, the majority of patients presented with fever (81.3%, 78/96) or cough (92.7%, 89/96), and almost one-third of patients (30.2%, 29/96) exhibited wheezing. The other 8 patients (8.3%, 9/96) also have exhibited fever, although this symptom was not recorded as the main complaint. Compared to mild cases, a significantly higher proportion of patients in the severe group exhibited wheezing symptoms (*P* = 0.001, 21.1% *vs* 56%). Twelve patients presented with dyspnea on admission, all of whom were categorized into the severe group. Furthermore, 11 severe cases were admitted to the Pediatric Intensive Care Unit during their hospitalization. The majority of patients exhibited two or more respiratory symptoms. A higher proportion of patients in the mild group presented with symptoms of both cough and fever (*P* = 0.003, 66.2% *vs* 32%). Patients with both cough and wheezing (*P* = 0.018, 5.6% *vs* 24.0%), as well as those exhibiting concomitant fever, cough, wheezing (*P* = 0.063, 12.7% *vs* 32.0%), were more severely ill. The respiratory rate and heart rate upon admission were significantly higher in severe patients compared to mild cases. Notably, severe patients experienced longer hospital stays, with a median duration of 8 days, in contrast to 6 days for mild patients (*P* = 0.000321). Clinical respiratory symptoms resolved or disappeared by the time of discharge in almost all patients, except for one critically ill patient with a high suspicion of hemophagocytic syndrome who ultimately dropped out of treatment.

### Coinfections with other causative agents based on tNGS

**Fig. 3:**
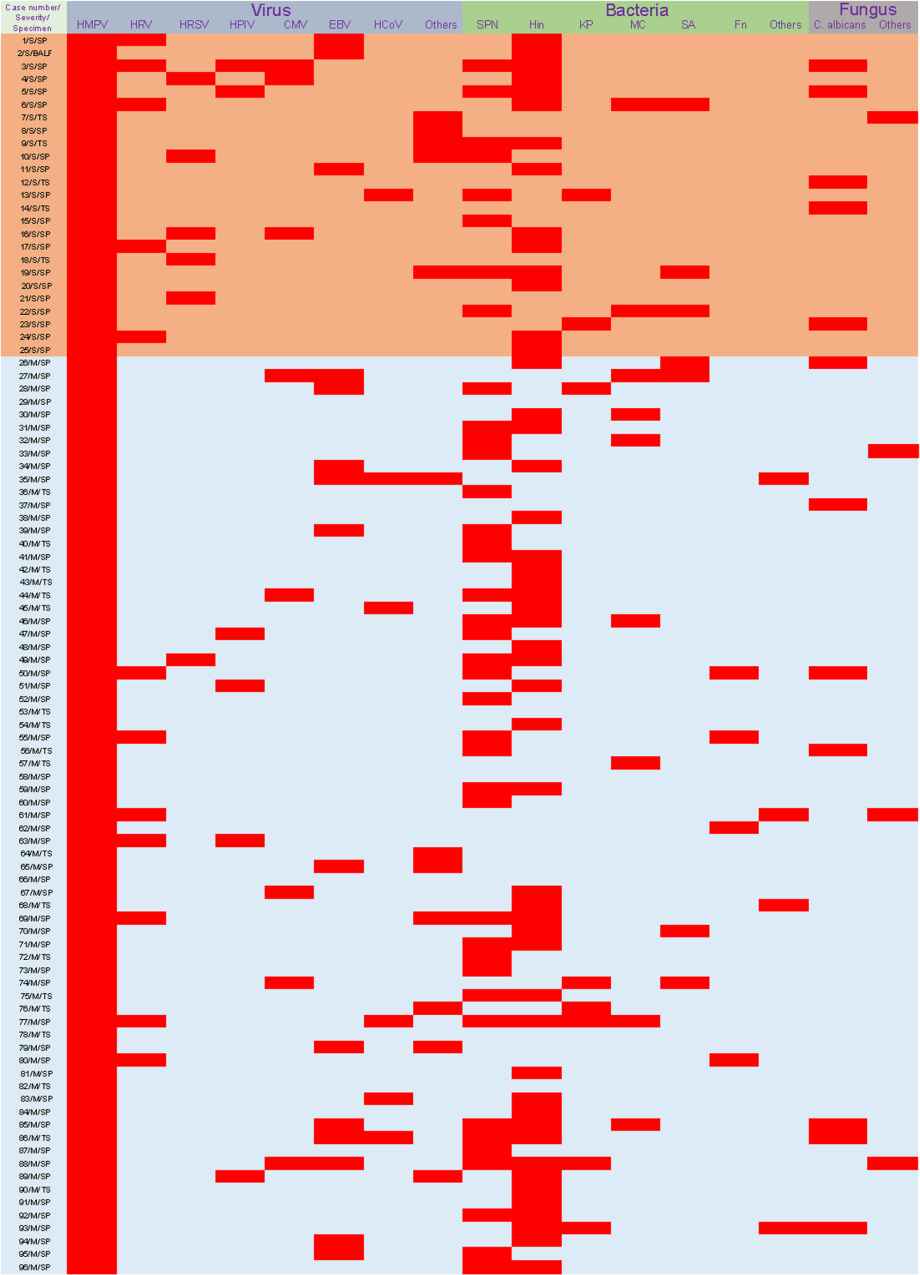
Coinfections in HMPV-infected patients. Red squares represent infection. Samples from case 44, 55 and 92 were subjected to multiple targeted sequencing of upper respiratory tract pathogens, including 105 pathogens. The others were subjected to targeted sequencing of multiple respiratory pathogens contained 198 pathogens. See Supplementary file 1 for more details of the targeted sequencing project. SP, sputum; TS, throat swab

Among the 96 HMPV-infected patients, 91 (94.8%) were coinfected with other causative agents (Table 2). Correspondingly, 5 patients were solely infected with HMPV, presenting symptoms of fever and coughing. Additionally, forty-three patients were infected with another virus. Coinfections of HMPV and EBV (15.6%, 15/96) or HRV type A (12.5%, 12/96) were the most common. The rate of HRSV coinfections (20%) was significantly higher in the severe group compared to the mild group (1.4%). Bacterial coinfections were identified in 74 patients, with Hin detected in 50 children (52.1%), SNP in 40 children (41.7%), MC in 9 children (9.4%), KP in 8 children (8.3%), and SA in 7 children (7.3%). Regarding fungal coinfections, *C. albicans* infection in the upper respiratory tract was the most prevalent. Further details about the pathogens infecting all patients are shown in Fig. 3.

**Table 2:** Mixed infection with other causative agents based on tNGS

	**All patients** **(No. = 96)**	**Mild pneumonia** **(No. = 71)**	**Severe pneumonia** **(No. = 25)**	***P* value**
**With mixed infection, No., (%)**	91 (94.8)	66 (93)	25 (100.0)	0.173
**With mixed virus infection, No., (%)**	43 (44.8)	25 (35.2)	18 (72)	0.001
EBV, No., (%)	15 (15.6)	12 (16.9)	3 (12)	0.752
HRV-A, No., (%)	12 (12.5)	7 (9.9)	5 (20)	0.168
CMV, No., (%)	8 (8.3)	5 (7.0)	3 (12)	0.425
HRSV, No., (%)	6 (6.3)	1 (1.4)	5 (20)	0.004
HPIV type3, No., (%)	6 (6.3)	4 (5.6)	2 (8)	0.649
HCoV, No., (%)	6 (6.3)	5 (7.0)	1 (4)	1.000
Other viruses, No., (%)	12 (12.5)	7 (9.9)	5 (20)	0.289
**With mixed bacterium infection, No., (%)**	74 (77.1)	55 (77.5)	19 (76)	0.881
SPN, No., (%)	40 (41.7)	32 (45.1)	8 (32)	0.346
Hin, No., (%)	50 (52.1)	36 (50.7)	14 (56)	0.816
KP, No., (%)	8 (8.3)	6 (8.5)	2 (8)	1.000
MC, No., (%)	9 (9.4)	7 (9.9)	2 (8)	1.000
SA, No., (%)	7 (7.3)^a^	4 (5.6)	3 (12)	0.372
Other bacteria, No., (%)	8 (8.3)	8 (11.3)	0 (0.0)	0.107
**With mixed fungus infection, No., (%)**	16 (16.7)	10 (14.1)	6 (24)	0.348
* C. albicans*, No., (%)	12 (12.5)	8 (11.3)	4 (16)	0.504
Other fungus, No., (%)	4 (4.2)	3 (4.2)	1 (4)	1.000

*P* values indicate the difference between pediatric patients with mild clinical type and those with severe clinical type

*Abbreviations: EBV* Epstein–Barr virus, *HRV-A* Human rhinovirus A, *CMV* Cytomegalovirus, *HRSV* Human respiratory syncytial virus, *HPIV* type3 Human parainfluenza virus type 3, *HCoV* Human coronavirus, *SPN* Streptococcus pneumoniae, *Hin* Haemophilus influenza, *KP* Klebsiella pneumoniae, *MC* Moraxella catarrhalis, *SA* Staphylococcus aureus, *C. albicans* Canidia albicans

^a^Gene of methicillin-resistant staphylococcal resistance was detected in two samples, one for each group

### Laboratory test findings

As shown in Table 3, severe patients exhibited a significantly lower count of EOS (*P* = 0.001), ESR count (*P* = 0.013), percentage of BASO (*P* = 0.013), percentage of EOS (*P* = 0.000273), and percentage of LYMPH (*P* = 0.000007), compared to mild group. Conversely, severe patients exhibited an increased percentage of NEUT (*P* = 0.000022), and a higher count of NEUT (*P* = 0.001), MONO (*P* = 0.04). Additionally, severe patients exhibited more evidence of multiple-organ damage compared to mild cases, as indicated by higher levels of unconjugated bilirubin (*P* = 0.038), alanine aminotransferase (*P* = 0.004), aspartate aminotransferase (*P* = 0.014), gamma-glutamyl transferase (*P* = 0.000004), lactate dehydrogenase (*P* = 0.004), and creatine kinase-MB (*P* = 0.035). In contrast, levels of conjugated bilirubin (*P* = 0.032), creatinine (*P* = 0.014) and uric acid (*P* = 0.04) decreased. Furthermore, several coagulation-related indices showed statistical significance between the two groups, including prolonged thrombin time, elevated prothrombin activity, decreased fibrinogen concentration, shortened prothrombin time and reduced international normalized ratio in severe group. Besides, decreased concentrations of Immunoglobulin G and Immunoglobulin A were observed in the severe group compared to the mild group. Other laboratory indices or organ damage biomarkers in the two groups were presented in Table 3.

**Table 3:** Laboratory findings of patients on admission

	**All patients** **(No. = 96)**	**Mild pneumonia** **(No. = 71)**	**Severe pneumonia** **(No. = 25)**	***P* value**
**Blood routine examination**	--	--	--	--
WBC count, median (IQR), No., 10^9/L,	7.4 (5.2, 9.9), 81	7.1 (5.1, 9.5), 61	8.8 (5.2, 13.2), 20	0.090
RBC count, median (IQR), No., 10^12/L	4.4 (4.2, 4.7), 81	4.4 (4.1, 4.7), 61	4.4 (4.3 4.5), 20	0.607
Hemoglobin, median (IQR), No., g/L	119.0 (113.0, 126.0), 81	120.0 (113.0, 126.0), 61	118.5 (109.0, 122.8), 20	0.261
PLT, median (IQR), No., 10^9/L,	292.0 (233.0, 387.0), 81	278.0 (216.0, 361.5), 61	327.5 (259.0, 406.5), 20	0.087
%NEUT, median (IQR), No., %	42.5 (31.4, 57.2), 81	37.0 (29.1, 50.0), 61	57.4 (45.6, 73.1), 20	0.000022
%LYMPH, median (IQR), No., %	49.8 (36.9, 59.9), 81	55.5 (42.9, 61.9), 61	36.9 (20.7, 43.9), 20	0.000007
%MONO, median (IQR), No., %	6.1 (4.7, 8.4), 81	6.0 (4.8, 7.8), 61	7.5 (4.2, 9.0), 20	0.446
%EOS, median (IQR), No., %	0.6 (0.0, 0.2), 81	0.8 (0.1, 1.8), 61	0.0 (0.0, 0.3), 20	0.000273
%BASO, median (IQR), No., %	0.2 (0.2, 0.4), 81	0.3 (0.2, 0.4), 61	0.2 (0.0, 0.3), 20	0.013
NEUT count, median (IQR), No., 10^9/L,	3.1 (1.6, 4.9), 81	2.9 (1.5, 3.8), 61	4.7 (3.0, 8.3), 20	0.001
LYMPH count, median (IQR), No., 10^9/L,	3.3 (2.2, 5.0), 81	3.6 (2.4, 5.1), 61	2.5 (1.6, 4.4), 20	0.064
MONO count, median (IQR), No., 10^9/L,	0.4 (0.3, 0.7), 81	0.4 (0.3, 0.5), 61	0.7 (0.4, 1.1), 20	0.040
EOS count, median (IQR), No., 10^9/L,	0.04 (0.00, 0.11), 81	0.06 (0.10, 0.12), 61	0.00 (0.00, 0.03), 20	0.001
BASO count, median (IQR), No., 10^9/L,	0.02 (0.01, 0.03), 81	0.02 (0.01, 0.03), 61	0.02 (0.00, 0.04), 20	0.409
CRP, median (IQR), No., mg/L	3.2 (0.5, 10.6), 81	3.4 (0.5, 10.4), 61	3.0 (0.5, 11.0), 20	0.894
ESR, median (IQR), No., mm/h	21.0 (9.0, 28.0), 72	22.0 (13.3, 30.0), 52	14.5 (3.0, 21.0), 20	0.013
**Coagulation profile**	--	--	--	--
Prothrombin time, median (IQR), No., S	11.5 (10.6, 12.0),43	11.7 (11.4, 12.3), 25	10.7 (10.4, 11.7), 18	0.013
Prothrombin activity, median (IQR), No., %	101.4 (96.3, 111.9), 43	99.3 (94.0, 103.1), 25	110.6 (99.3, 114.8), 18	0.013
International normalized ratio, median (IQR), No	1.0 (0.9, 1.0), 43	1.0 (1.0, 1.1), 25	0.9 (0.9, 1.0), 18	0.013
Activated partial thromboplastin time, median (IQR), No., S	31.4 (26.8, 33.9), 42	31.4 (29.0, 33.6), 25	30.8 (25.1, 34.3), 17	0.405
Fibrinogen concentration, median (IQR), No., g/L	3.1 (2.4, 3.7),43	3.4 (3.0, 4.0), 25	2.4 (1.7, 3.1), 18	0.000355
Thrombin time, median (IQR), No., S	15.4 (14.5, 16.2), 43	14.9 (14.3, 15.7), 25	15.8 (15.2, 17.4), 18	0.008
**Blood biochemical parameters**	--	--	--	--
Total bilirubin, median (IQR), No., umol	4.8 (3.6, 6.6), 91	4.7 (3.4, 6.2), 67	5.1 (4.0, 7.7), 24	0.253
Conjugated bilirubin, median (IQR), No., umol	1.7 (1.2, 2.2), 91	1.8 (1.3, 2.2), 67	1.1 (0.7, 2.2), 24	0.032
Unconjugated bilirubin, median (IQR), No., umol	3.3 (2.1, 4.5), 91	2.9 (1.9, 4.3), 67	4.1 (2.6, 5.0), 24	0.038
ALT, median (IQR), No., U/L	17.0 (12.8, 21.0), 91	15.6 (12.1, 19.5), 67	19.5 (17.0, 27.8), 24	0.004
AST, median (IQR), No., U/L	37.0 (30.1, 43.6), 91	36.6 (29.5, 42.1), 67	42.5 (35.6, 49.6), 24	0.014
ALP, median (IQR), No., U/L	169.0 (141.7, 214.6), 91	169.4 (142.2, 209.9), 67	157.1 (123.0, 233.4), 24	0.903
GGT, median (IQR), No., U/L	12.6 (11.0, 14.9), 89	12.0 (10.5, 13.2), 65	16.7 (13.1, 29.6), 24	0.000004
LDH, median (IQR), No., U/L	313.8 (272.8, 393.0), 90	297.5 (271.8, 369.8), 66	384.0 (313.4, 425.8), 24	0.004
α-HBBDH, median (IQR), No., U/L	222.5 (199.3, 253.8), 64	222.0 (199.5, 253.5), 53	223.0 (198.0, 269.0), 11	0.845
CK, median (IQR), No., U/L	83.5 (54.7, 109.3), 90	86.5 (56.0, 111.3), 66	63.0 (45.8, 102.0), 24	0.096
CK-MB, median (IQR), No., U/L	20.0 (16.0, 27.2), 90	18.5 (15.3, 25.3), 66	25.0 (16.0, 38.3), 24	0.035
Total protein, median (IQR), No., g/L	62.2 (60.3, 65.3), 91	62.2 (60.3, 65.1), 67	62.2 (60.0, 66.3), 24	0.787
Albumin, median (IQR), No., g/L	40.5 (38.6, 42.5), 91	40.6 (38.5, 42.8), 67	40.2 (38.7, 42.1), 24	0.627
Globulin, median (IQR), No., g/L	22.6 (19.7, 24.3), 91	22.8 (19.5, 24.4), 67	22.4 (20.1, 24.2), 24	0.900
Urea, median (IQR), No., mmol	2.9 (2.2, 3.6), 90	2.9 (2.3, 3.5), 66	2.7 (1.8, 4.0), 24	0.902
Creatinine, median (IQR), No., umol	22.1 (17.9, 29.4), 90	23.2 (19.1, 29.8), 66	18.5 (15.1, 25.5), 24	0.014
Uric acid, median (IQR), No., umol	218.5 (184.2, 268.0), 90	229.0 (194.7, 270.0), 66	196.1 (133.3, 245.9), 24	0.040
Kalium, median (IQR), No., mmol	4.3 (4.0, 4.7), 76	4.3 (4.0, 4.7), 60	4.3 (4.1, 4.7), 16	0.990
Sodium (Na), median (IQR), No., mmol	139.0 (137.3, 140.0), 76	139.2 (137.5, 140.2), 60	138.3 (136.5, 139.5), 16	0.221
Chlorine, median (IQR), No., mmol	104.8 (102.4, 106.7), 76	105.1 (102.5, 107.0), 60	104.3 (101.7, 106.1), 16	0.238
Calcium, median (IQR), No., mmol	2.4 (2.3, 2.4), 76	2.4 (2.3, 2.4), 60	2.3 (2.1, 2.5), 16	0.707
Phosphorus, median (IQR), No., mmol	1.5 (1.4, 1.7), 40	1.5 (1.3, 1.7), 26	1.6 (1.4, 1.7), 14	0.435
Magnesium, median (IQR), No., mmol	0.9 (0.9, 1.0), 37	0.9 (0.9, 1.0), 25	1.0 (0.9, 1.0), 12	0.884
**Immunology index**	--	--	--	--
Ig E, median (IQR), No., ng/ml	141.6 (48.5, 433.3), 81	147.3 (50.5, 562.0), 63	125.2 (38.1, 314.0), 18	0.450
Ig G, median (IQR), No., g/L	7.5 (5.7, 9.2), 82	7.7 (6.3, 9.3), 62	5.7 (5.1, 8.6), 20	0.021
Ig M, median (IQR), No., g/L	1.0 (0.8, 1.3), 82	1.1 (0.8, 1.3), 62	1.0 (0.7, 1.3), 20	0.646
Ig A, median (IQR), No., g/L	0.6 (0.4, 1.0), 82	0.7 (0.5, 1.2), 62	0.4 (0.4, 0.6), 20	0.007
Complement C3, median (IQR), No., g/L	1.1 (1.0, 1.3), 82	1.1 (1.0, 1.3), 62	1.1 (0.9, 1.2), 20	0.087
Complement C4, median (IQR), No., g/L	0.3 (0.3, 0.4), 82	0.3 (0.3, 0.4), 62	0.3 (0.2, 0.3), 20	0.118

*P* values indicate the difference between pediatric patients with mild clinical type and those with severe clinical type. No. is the total number of patients with available data

*Abbreviations: WBC* white blood cell, *RBC* red blood cell, *PLT* platelet count, *%NEUT* neutrophil percentage, *LYMPH* lymphocyte, *MONO* monocyte, *EOS* eosinophilic granulocyte, *BASO* basophilic granulocyte, *CRP* C-reactive protein, *ESR* erythrocyte sedimentation rate, *ALT* alanine aminotransferase, *AST* aspartate aminotransferase, *ALP* alkaline phosphatase, *GGT* gamma-glutamyl transferase, *LDH* lactate dehydrogenase, *CK* creatine kinase, *CK-MB* creatine kinase-MB, α-HBBDH α-hydroxybutyrate dehydrogenase, *Ig* immunoglobulin

### Inflammatory responses in HMPV-infected patients

**Fig. 4:**
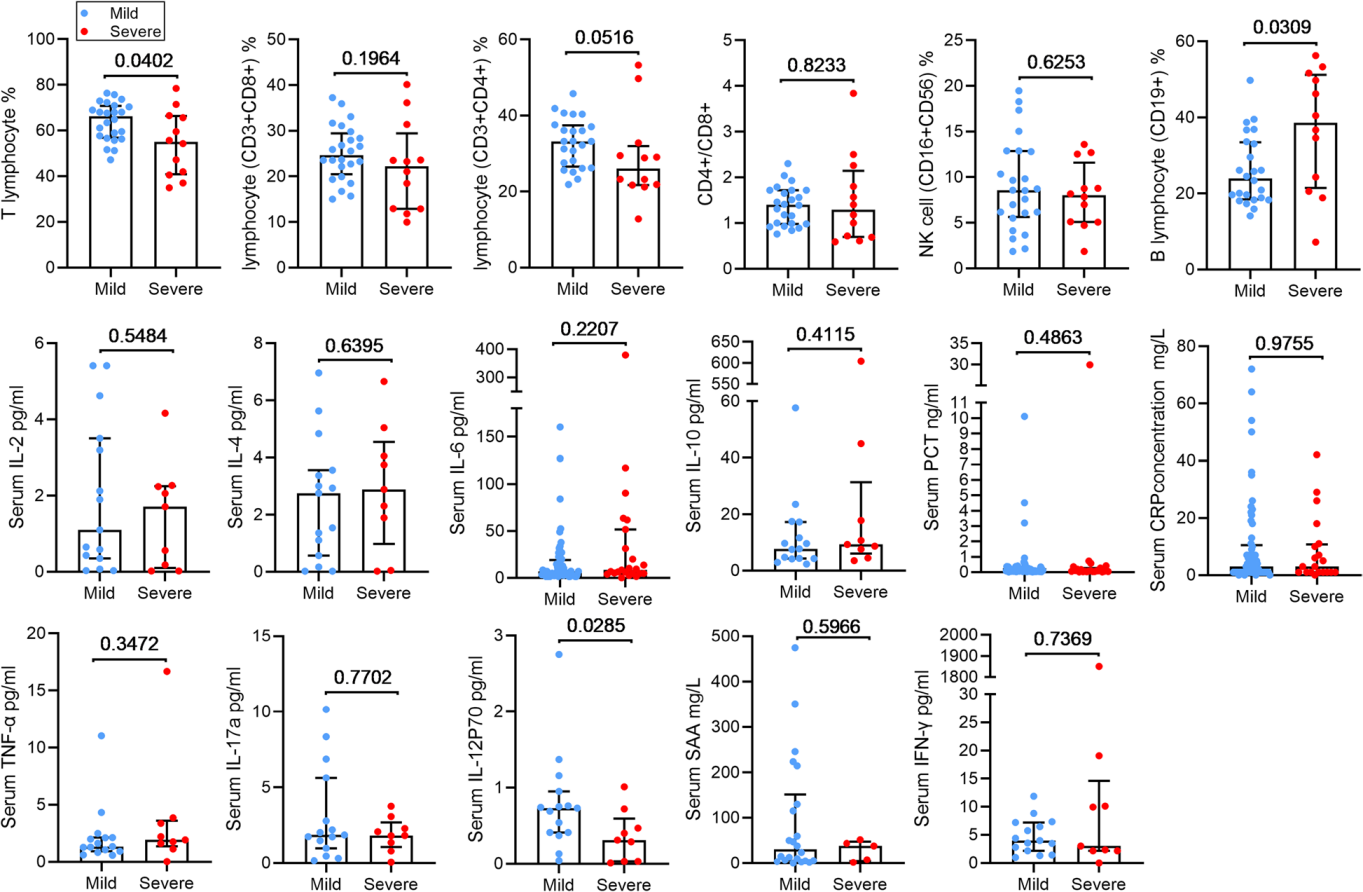
Inflammatory response in HMPV-infected patients. PCT, procalcitonin; SAA, serum amyloid A; The histogram represents the median and the whiskers represents the IQR

To further elucidate the immune response, we analyzed the changes in lymphocyte subpopulations, serum cytokines, and chemokines levels were also analyzed (Fig. 4). We observed higher levels of cytokines, including interleukin (IL)-2, IL-4, IL-6, IL-10, tumor necrosis factor (TNF) -α, and SAA in severe patients compared to mild patients, although these differences did not reach statistical significance. Conversely, the levels of interferon (IFN)-γ, IL-17a, IL-12p70, were decreased in the severe patients, but only the lower levels of IL-12p70 exhibited statistical significance between the two groups (*P* = 0.029). Regarding immune cells, the decline in lymphocytes percentage was more pronounced in severe patients (*P* = 0.0402), while the percentage of B lymphocyte was significantly elevated (*P* = 0.0309). There was no significant difference in the percentage of CD4 + T cells, CD8 + T cell, and NK cell, as well as the ratio of CD4 + T cells to CD8 + T cells between the two groups. Notably, severe patients exhibited more pronounced aggravated inflammatory responses, lymphopenia, and multiple-organ damage compared to those in mild cases.

## Dynamic change of several altered indicators

**Fig. 5:**
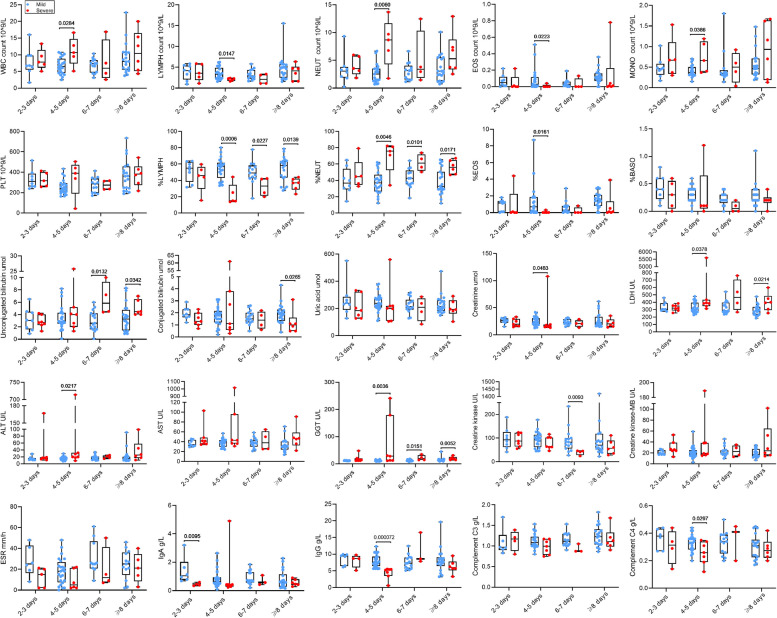
Subgroup analyses of laboratory indicators. The box plots show the medians (middle line) and first and third quartiles (boxes), and the whiskers show range of the measured values. *Abbreviations*: *WBC* white blood cell, *PLT* platelet count, *NEUT* neutrophil, *LYMPH* lymphocyte, *MONO* monocyte, *EOS* eosinophilic granulocyte, *BASO* basophilic granulocyte, *LDH* lactate dehydrogenase, *ALT* alanine aminotransferase, *AST* aspartate aminotransferase, *GGT* gamma-glutamyl transferase, *ESR* erythrocyte sedimentation rate, *Ig* immunoglobulin

All subjects were stratified into different subsets based on the duration from illness onset to hospitalization, including subsets of 2–3, 4–5, 6–7, and ≥ 8 days. The altered indicators (*P* ≤ 0.2) were analyzed within these subgroups. As shown in Fig. 5, there was no statistically significant difference in PLT, percentage of BASO, uric acid, AST, CK-MB, ESR and C3 between the two groups. Obviously, most indicators exhibited significant changes after 4–5 days of illness onset. Compared to mild patients, severe cases exhibited significantly increased WBC count, NEUT count, MONO count, percentage of NEUT, and LDH, ALT, GGT in the peripheral blood at 4–5 days after illness onset. Conversely, LYMPH count, EOS count, percentage of LYMPH, percentage of LYMPH, and creatinine, IgG, and C4 in the serum were significantly reduced during the same period. The changes in percentage of LYMPH and NEUT remained consistent after 4–5 days after illness onset. Figure 5 showed specific changes in other indicators.

### Radiologic findings

**Fig. 6:**
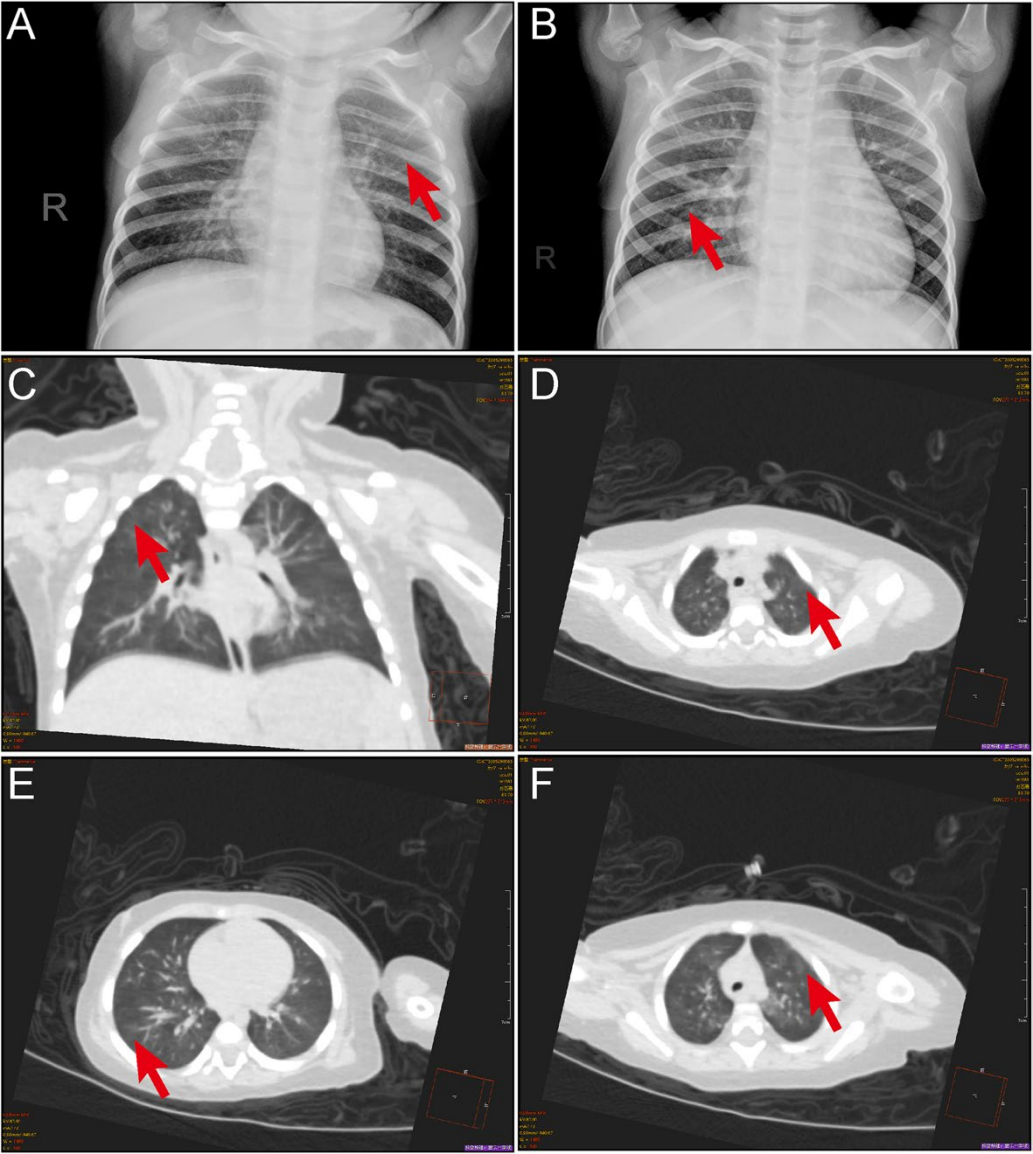
Pulmonary manifestations of HMPV infection. **A** CT scan of 1-year-old girl showing multiple ground-glass opacities in both lungs (arrow). **B** Chest radiograph of a 4-year-old girl displaying multiple ground-glass opacities (arrow). **C-F** Chest CT scans from a 7-month-old girl showing focal ground-glass opacities and stripes coexisting in the lung (arrows)

As mentioned previously, nearly all patients were coinfected with other causative agents, while only 5 patients were solely infected with HMPV. Among these, imaging examinations were conducted for three of the five patients. Despite presenting with mild clinical symptoms such as fever and cough, all three patients exhibited abnormal radiological findings, including focal ground-glass opacities and/or stripe shadows (Fig. 6).

## Discussion

The etiological significance of HMPV in ARTI has been the focus of increasing attention worldwide [3]. HMPV infection among hospitalized children with ARTIs experienced a significant decrease during COVID-19 pandemic [14]. However, with the relaxation of COVID-19 mitigation measures, HMPV transmission has surpassed pre-epidemic levels [10]. The immunity debt [9] and the alterations in prevalent genetic subtypes [14] following the COVID-19 pandemic have raised our concerns. Hence, this study aimed to evaluate the epidemiological and clinical characteristics of HMPV infections in hospitalized children in Henan, China from April 29 to June 5, 2023.

In this study, we observed that the majority (87.5%) of infected children were under 5 years, with more than half (65.63%) being under 3 years old. Previous study has indicated that the hospital admission rate for HMPV-associated ARTI is notably higher among infants compared to older children, with approximately 58% of hospital admissions in children under 5 years occurring within the first year of life [4, 21]. The elevated burden on infants may be attributed to the immaturity of their immune systems and the decline of maternal antibodies during the first months of life [22].

Upon admission, we observed that patients presenting with concurrent cough and wheezing, as well as those with simultaneous fever, cough, and wheezing, tended to be more severely ill. Generally, HMPV-associated respiratory diseases manifest along a spectrum from mild fever and cough to severe bronchiolitis and pneumonia [13, 15]. A study involving hospitalized children in Beijing similarly reported that the majority of patients presented with cough and fever [14]. Wheezing is a common manifestation in children with HMPV infection, often progressing to life-threatening bronchiolitis and pneumonia [23]. A study has indicated that 13.0% ~ 60.7% of children infected with HMPV experience recurrent wheezing or receive a diagnosis of asthma [24]. However, distinguishing HMPV-associated pneumonia from infection caused by other pathogens based solely on clinical features remains challenging [25–27]. Several respiratory pathogens, including HMPV, exhibit similar incidence rates and clinical characteristics, thereby posing a diagnostic challenge for attending physicians.

A previous study revealed that HMPV was the third most common virus associated with coinfection [28]. Viral cocirculation can lead to competition among viruses, whereby the stronger virus may survive and/or mutate, potentially resulting in higher mortality and morbidity rates [29]. In our study, we identified 43 patients who were coinfected with another virus, with coinfections of HMPV with EBV (15.6%) or HRV type A (12.5%) being common. EBV infects over 95% of the global population and is often asymptomatic, typically occurring at a young age [30]. Unlike EBV, HRV type A is responsible for more than 50% of upper respiratory tract infections globally and it is the most common virus associated with wheezing in children aged between one and two years [31]. Previous studies have identified influenza, HRSV, adenoviral, and human bocavirus as the most common respiratory viruses involved in coinfections [14, 15, 23, 32]. Our results findings diverged from previously published data, which could be attributed to the seasonality of different viruses [33], variations in sample types, and differences in sample sizes among studies. In our study, the rate of HRSV coinfections was significantly higher in the severe group compared to the mild group. Previous research has indicated that dual infections with HMPV and RSV are associated with severe bronchiolitis and an increased risk of Intensive Care Unit admission for mechanical ventilation [34]. Additionally, coinfections with bacterial pathogens often lead to a more severe course and increased mortality. While HMPV-associated pneumonia is generally less severe than bacterial pneumonia, it can be as severe as or even more severe than infections caused by other common pathogens [27]. In our study, Hin and SNP were the two most common bacterial coinfections, accounting for 52.1% and 41.7%, respectively. Therefore, further data are necessary to elucidate the cumulative clinical effects on disease severity resulting from coinfecting with other respiratory pathogens.

Significant alterations in several indicators among severe patients were notably observed at 4–5 days after the onset of illness, marking a critical time point for disease progression, and necessitating timely decisions regarding treatment interventions. Therefore, we should pay more attention to the diagnosis and intervention in the early stage of the disease. In our study, we observed a lower level of IL-12p70 in severe patients. However, previous research suggested that HMPV failed to induce production of IL-12p70 in BALF in a mouse model [35]. In terms of immune cells, we observed a more pronounced decline in the lymphocyte percentage among severe patients, while the percentage of B lymphocyte was significantly elevated. In other words, the function of the specific cellular immunity was decreased, while the function of humoral immune was increased. Nevertheless, the decreased concentrations of Immunoglobulin G and Immunoglobulin A in the severe group contradicted these results. In fact, interpreting these results is often challenging due to the complexities of the disease and the involvement of mixed infections. In clinical practice, patients with single HMPV infections are rare, emphasizing the necessity for further research on patients with mixed infections.

This study also had several limitations. Firstly, our focus was primarily on the epidemiological and clinical features of HMPV positive cases. Thus, other common respiratory pathogens were not excluded. Subsequently, our results more reflect the cumulative clinical effects of coinfection. Secondly, children with more severe symptoms are more likely to receive hospital care, which may affect our understanding of the clinical features of HMPV-positive patients. Despite these shortcomings, our results offer a valuable insight into the epidemiological and clinical characteristics of hospitalized patients with HMPV infection in Henan, China.

## Conclusion

Our study elucidated important epidemiological and clinical characteristics of HMPV infection in Henan, China. These findings hold potential significance for informing policy development aimed at the prevention and control of HMPV infection. Additionally, our results may provide important insights for guiding HMPV research efforts in the post- COVID-19 pandemic era.

### Supplementary Information

**Supplementary Material 1.**

**Supplementary Material 2.**

## Data Availability

No datasets were generated or analysed during the current study.

## Abbreviations

ARTI: Acute respiratory tract infections
ALT: Alanine aminotransferase
AST: Aspartate aminotransferase
ALP: Alkaline phosphatase
α-HBBDH: α-hydroxybutyrate dehydrogenase
BALF: Bronchoalveolar lavage fluid
BASO: Basophilic granulocyte
CK: Creatine kinase
CK-MB: Creatine kinase-MB
CRP: C-reactive protein
CMV: Cytomegalovirus
C. albicans: Canidia albicans
EOS: Eosinophilic granulocyte
ESR: Erythrocyte sedimentation rate
EBV: Epstein–Barr virus
GGT: Gamma-glutamyl transferase
HRV-A: Human rhinovirus A
HRSV: Human respiratory syncytial virus
HPIV type3: Human parainfluenza virus type 3
HCoV: Human coronavirus
Hin: Haemophilus influenza
HMPV: Human metapneumovirus
IQR: Interquartile range
Ig: Immunoglobulin
IFN: Interferon
IL: Interleukin
KP: Klebsiella pneumoniae
LYMPH: Lymphocyte
LDH: Lactate dehydrogenase
MONO: Monocyte
MC: Moraxella catarrhalis
NEUT: Neutrophil
PLT: Platelet count
PCT: Procalcitonin
RBC: Red blood cell
SPN: Streptococcus pneumoniae
SAA: Serum amyloid A
SA: Staphylococcus aureus
TNF: Tumor necrosis factor
tNGS: Targeted Next Generation Sequencing
WBC: White blood cell
