# Supplementary figures and images for: Clinical and epidemiological characteristics of 96 pediatric human metapneumovirus infections in Henan, China after COVID-19 pandemic: a retrospective analysis
Source: Virol J. 2024 Apr 30;21:100. doi: 10.1186/s12985-024-02376-0 (PMC11059775; doi:10.1186/s12985-024-02376-0)

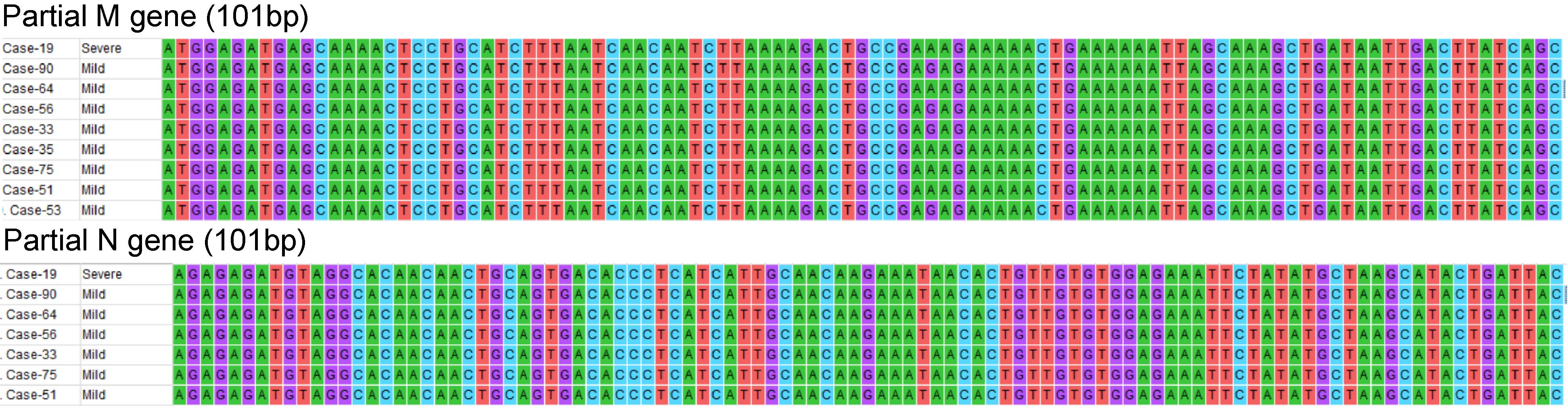

Supplement: Supplementary file 2 — Supplementary Material 2. [file 12985_2024_2376_MOESM2_ESM.tif]
